# Simultaneous Quantification of Nine New Furanocoumarins in Angelicae Dahuricae Radix Using Ultra-Fast Liquid Chromatography with Tandem Mass Spectrometry

**DOI:** 10.3390/molecules22020322

**Published:** 2017-02-20

**Authors:** Lei Zhang, Wei Wei, Xiu-Wei Yang

**Affiliations:** State Key Laboratory of Natural and Biomimetic Drugs, Department of Natural Medicines, School of Pharmaceutical Sciences, Peking University Health Science Center, Peking University, No. 38, Xueyuan Road, Haidian District, Beijing 100191, China; zhangyutian0619@163.com (L.Z.); gg-993@163.com (W.W.)

**Keywords:** furanocoumarin, Angelicae Dahuricae Radix, UFLC-MS/MS, simultaneous quantification

## Abstract

A series of new furanocoumarins with long-chain hydrophobic groups, namely andafocoumarins A–H and J, have been isolated from the dried roots of *Angelica dahurica* cv. Hangbaizhi (Angelicae Dahuricae radix) in our previous study, among which andafocoumarins A and B were demonstrated to have better anti-inflammatory activity than the positive controls. In this work, a sensitive, accurate, and efficient ultra-fast liquid chromatography coupled with triple quadrupole mass spectrometer (UFLC-MS/MS) method was developed and validated for simultaneous quantification of above-mentioned nine compounds in four cultivars of Angelicae Dahuricae Radix. Chromatographic separation was performed on a Kinetex 2.6u C18 100 Å column (100 × 2.1 mm, 2.6 µm). The mobile phases were comprised of acetonitrile and water with a flow rate of 0.5 mL/min. Using the established method, all components could be easily separated within 12 min. With the multiple reaction monitor mode, all components were detected in positive electrospray ionization. The method was validated with injection precision, linearity, lower limit of detection, lower limit of quantification, precision, recovery, and stability, respectively. The final results demonstrated that the method was accurate and efficient, which could be used to simultaneously quantify the nine andafocoumarins in Angelicae Dahuricae Radix. The results also indicated that in different batches of Angelicae Dahuricae Radix, some of the andafocoumarins were significantly different in terms of content.

## 1. Introduction

The traditional Chinese medicine “Angelicae Dahuricae Radix” (ADR), prepared from the roots of *Angelica dahurica* cv. Hangbaizhi, *A. dahurica* cv. Chuanbaizhi, *A. dahurica* cv. Yubaizhi, and *A. dahurica* cv. Qibaizhi, has been prescribed for the treatment of headache, toothache, abscess, nose congestion, acne, and furunculosis [1,2]. Furanocoumarins are one kind of important bioactive compounds naturally existing in ADR. Thus far, many furanocoumarins including dimeric furanocoumarins [3,4] have been isolated from ADR with beneficial pharmacological effects such as anti-dementia [5], anti-proliferative [6], anti-oxidative [7,8], anti-inflammatory [3], anti-microbial [9], anti-hypertensive [10], and anti-cancer properties [11,12], as well as inhibiting advanced glycation end products (AGEs) [13]. Imperatorin, isoimperatorin, oxypeucedanin hydrate, oxypeucedanin, xanthotoxol, xanthotoxin, and psoralen are considered as the major furanocoumarins in ADR according to our experiments and reports in the literature [14]. Many studies in recent years have focused on the quantitative analysis of furanocoumarins with high performance liquid chromatography-ultraviolet (HPLC-UV) and high-performance liquid chromatography coupled with tandem mass spectrometry (LC-MS/MS) methods [14,15,16,17,18,19,20,21,22]. When compared with HPLC-UV, the LC-MS/MS method has generated increased interest as it has high sensitivity and selectivity. With the help of a more efficient chromatographic column and a MS detector, LC-MS/MS is well qualified for the determination of trace components and for pharmacokinetic analysis.

In our previous study [23], a series of new furanocoumarins with short- and long-chain hydrophobic groups were isolated and identified from the roots of *A. dahurica* cv. Hangbaizhi. Pharmacological assays indicated that some of them possessed better anti-inflammatory activity than that of the positive controls. Therefore, it is important to develop an evaluation method for the quality control of these components in ADR.

**Figure 1:**
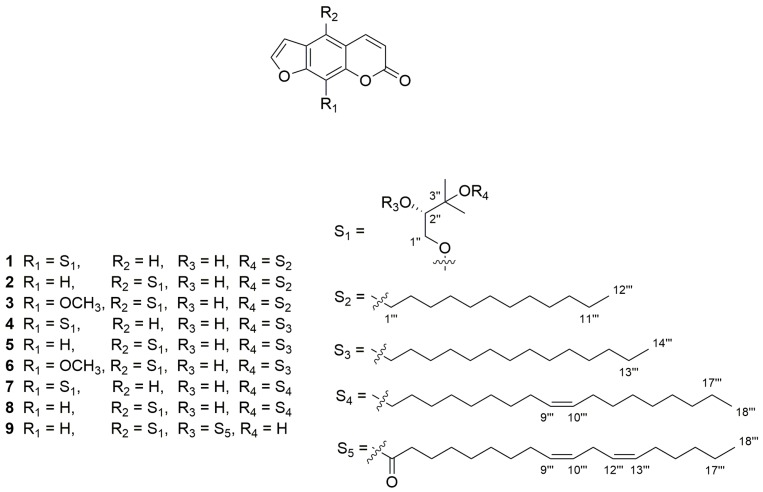
The structures of nine furanocoumarins isolated from Hangbaizhi collected from the Yangtou Village of Shenze Township in Pan’an county of Zhejiang province of China. From **1** to **9**, they are andafocoumarins A, B, C, D, E, F, G, H, and J.

Herein, a rapid and efficient LC-MS/MS method was developed and validated. Using this method, nine components (chemical structures are shown in Figure 1) could be quantitatively determined in a short time with good resolution and sensitivity. This is the first report of the simultaneous analysis and comparison of nine furanocoumarins in different cultivars of ADR and the results are anticipated to provide useful information for the further development of these compounds.

## 2. Results and Discussion

### 2.1. Optimization of Extraction Conditions

With the established LC-MS/MS method, the extraction conditions were optimized. The extraction method, extraction solvent, and extraction time were investigated and the results indicated these nine components could be completely extracted both by refluxing and ultrasonic methods. Considering the efficiency of operation, the ultrasonic method was selected as the final extraction method. Subsequently, considering the hydrophobic groups in these compounds, methanol (MeOH), diethyl ether, and acetone were compared to determine the extraction solvent. The result showed that all the solvents were suitable for extraction, and MeOH was chosen as the final solvent because no more procedures were needed prior to injection into the LC-MS/MS. Finally, to determine the optimal extraction time, 0.5 g samples were extracted with 20 mL of MeOH by the ultrasonic method for 30 and 60 min, respectively. As a result, these compounds could be completely extracted within 30 min.

### 2.2. Optimization of the LC-MS/MS Conditions

The optimal MS parameters for each component were obtained by direct injection of the standard solution into the mass spectrometer, respectively. Finally, in positive electrospray ionization (ESI) mode, most of the components responded better than in the negative ESI mode. Meanwhile, to increase the sensitivity and specificity, some other detection parameters such as Q1 Pre Bias (voltage promotes the ionization of the precursor ion), Q3 Pre Bias (voltage promotes the ionization of the product ion), and collision energy were optimized as well.

**Figure 2:**
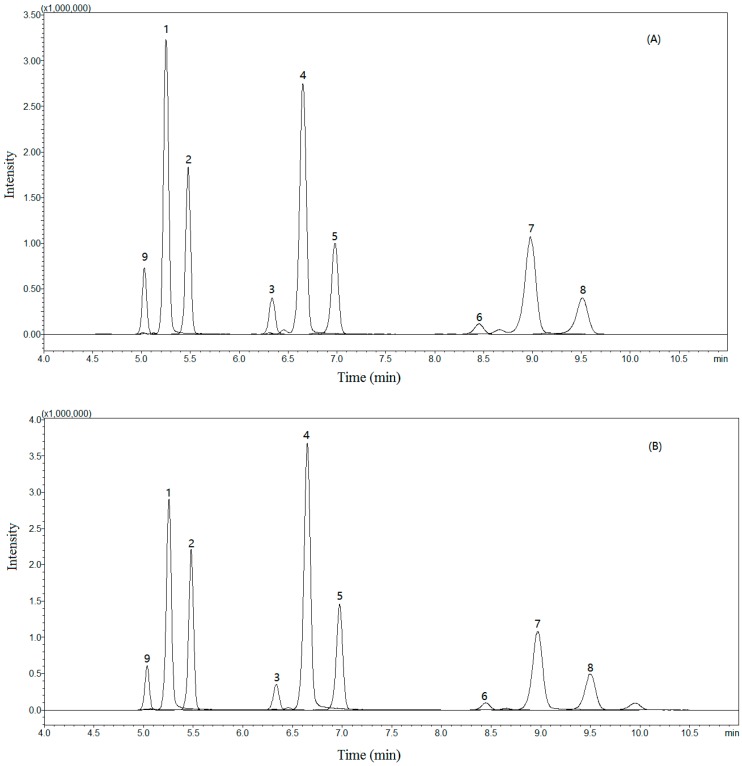
Typical total-ion multiple reaction monitor (MRM) chromatograms of the sample solution (**A**) and standard solution (**B**) obtained in positive-ion mode. Andafocoumarin A (**1**), andafocoumarin B (**2**), andafocoumarin C (**3**), andafocoumarin D (**4**), andafocoumarin E (**5**), andafocoumarin F (**6**), andafocoumarin G (**7**), andafocoumarin H (**8**), and andafocoumarin J (**9**).

The chromatographic conditions including solvent composition and gradient elution were also optimized to shorten the analysis time and maximize the response of the compounds. Through several trials, acetonitrile (ACN) and water (H_2_O) were chosen as the mobile phases and with a Kinetex 2.6u C18 100 Å column (100 mm × 2.1 mm i.d.; 2.6 μm), these nine components could be well separated and detected in different channels within 12 min without any interference. The typical chromatograms are shown in Figure 2.

### 2.3. Assay Validation

#### 2.3.1. Injection Precision, Calibration Curves, Lower Limit of Detection and Lower Limit of Quantification

The injection precision, linearity, lower limit of detection (LLOD), lower limit of quantification (LLOQ), precision, extraction recovery, and stability of the method were fully validated. The injection precision was determined by replicated injection of the same sample six times. The results showed that the relative standard deviation (RSD) of retention times and peak areas were lower than 0.35% and 3.71%, respectively.

The linearity of the plot of concentration (*x*, µg/mL) for each coumarin against the peak area (*y*) was investigated. As a result, the standard calibration curves showed good correlation coefficients (*r* > 0.996) of all the components. The detailed results are given in Table 1.

In general, a detector signal-to-noise ratio of 3:1 was denoted as the LLOD and 10:1 as the LLOQ, respectively. The minimum concentration of the linearity solution was used and diluted consecutively to obtain the LLOD and LLOQ of the nine components. The final results are also shown in Table 1.

#### 2.3.2. Precision and Stability

The intra- and inter-day precision of the method were determined by measuring the nine components in six replicates in a single day and by duplicating the operation over three consecutive days. Finally, the RSD of the intra- and inter-day precision were calculated and the results are presented in Table 2.

The stability of the standard and sample solutions was tested at normal temperature (25 °C) over a period of 24 h. The RSD of the peak areas were calculated and the results are listed in Table 3, which indicate that the components were basically stable for 24 h.

#### 2.3.3. Extraction Recovery

The extraction recoveries of the nine components were determined by measuring the samples at three concentrations with each concentration performed in three replicates. As can be clearly seen in Table 4, all the components at high, medium, and low levels had mean recoveries above 86.12% and RSD values below 6.56%.

### 2.4. Quantitative Determination of the Nine Analytes in Angelicae Dahuricae Radix

With the validated method, the contents of components **1**–**9** in different cultivars of ADR were accurately determined and calculated with the regression equations. For each batch of the crude drug, two parallel samples were prepared and tested. The detailed results are shown in Table 5.

LC-MS/MS has enormous advantages in the simultaneous quantitative analysis of multiple components, especially those in trace amounts. In this work, a LC-MS/MS method was successfully established and applied to quantify and compare nine furanocoumarins. As we could see, the total amounts of these nine coumarins fell in the range between 89.41–1022.57 µg/g. As mentioned above, it is worth noting that components **1** and **2** were two important constituents with anti-inflammatory activity and their contents were relatively high in the roots of *A. dahurica* cv. Hangbaizhi and *A. dahurica* cv. Chuanbaizhi. In addition, it was clearly shown that the contents of some components had evident variances in different cultivars or even within the same cultivars of ADR.

Recent studies have focused on the multi-component quantification and pharmacokinetics of the major bioactivity constituents, namely coumarins, which provide a lot of data for better application of ADR. The nine new compounds isolated from the roots of *A. dahurica* cv. Hangbaizhi in our previous study all had long-chain hydrophobic groups and three pairs of them are isomers (components **1** and **2**, **4** and **5**, and **7** and **8**) [23]. Therefore, the extraction and analysis method for simultaneous quantification is very hard to establish. Heating reflux was originally used as the extraction method and the ADR sample was extracted with 40-fold ethanol 3 times (2 h each). After being measured with LC-MS/MS, we could conclude that the nine compounds were almost completely extracted during the first reflux process (≥95%). However, obviously, the process was rather complex. Many previous studies [16,20,22] selected ultrasonic extraction because of its high efficiency. Considering the long-chain hydrophobic groups, MeOH, acetone, and diethyl ether were all used to compare the extraction efficiency. Through a series of tests and comparisons, 20-fold MeOH and a 30 min ultrasonic extraction were confirmed as the best extraction conditions for these nine components.

The resolution of peaks, determination of trace components, and analysis time are important indexes when establishing a chromatographic separation. In this study, a Kinetex 2.6u C18 100 Å column (Phenomenex Inc., Torrance, CA, USA) and MS detector (Shimadzu Corp., Kyoto, Japan) were used because they afforded sufficient separation and quantification of the nine components. Gradient elution was applied to satisfy the different polarity of these constituents. Through optimization, these nine furanocoumarins could be well eluted and separated within 12 min. It is hard to get the same effect with the LC-UV method. The LC-MS/MS method was quite sensitive, accurate, and efficient, and it is suitable for the determination of the nine novel furocoumarins in ADR.

## 3. Experimental Section

### 3.1. Plant Materials

Twenty batches of ADR (Table 6) from four different cultivars were collected. The species were identified by Prof. Xiu-Wei Yang of the School of Pharmaceutical Sciences, Peking University Health Science Center, Peking University. Voucher specimens were deposited at the State Key Laboratory of Natural and Biomimetic Drugs (Peking University, Beijing, China). The samples were stored at room temperature (25 °C) until they were used for analysis.

### 3.2. Chemicals, Reagents, and Standards

MeOH and ACN were of LC–MS grade from Fisher Scientific (Pittsburgh, PA, USA). All other chemicals used were available as products of at least analytical grade. Deionized H_2_O (18 MΩ/cm) was generated in-house using a Milli-Q System from the Millipore Corporation (Billerica, MA, USA). Reference standards, andafocoumarins A (**1**), B (**2**), C (**3**), D (**4**), E (**5**), F (**6**), G (**7**), H (**8**), and J (**9**) (as shown in Figure 1) were isolated and identified in our previous report [23]. The individual purity of each standard was confirmed over 95% according to Nuclear Magnetic Resonance (NMR) and LC–MS coupled with diode-array detector (DAD) analysis.

### 3.3. Preparation of Angelicae Dahuricae Radix Extracts

The ADR samples were pulverized into powder (40 mesh). The accurately weighed powder (0.5 g) was added into a conical flask with a cover and was ultrasonically extracted (40 kHz, 200 W) with 20 mL of MeOH for 30 min. The extracted solution was then filtered through a 0.22 μm Millipore filter prior to injecting into the LC-MS/MS system and the sample volume injected was set at 1 μL.

### 3.4. Preparation of Standard Solutions

Stock solutions (each 1 mg/mL) of the nine components were independently prepared by dissolving accurately weighed reference substances in MeOH. Working solutions for the calibration curves of the nine components were prepared by appropriate dilution of the stock solution with MeOH.

### 3.5. LC-MS/MS Analysis

The ultra-fast liquid chromatography coupled with a triple quadrupole mass spectrometer (UFLC-MS/MS) 8050 system (Shimadzu Corp., Kyoto, Japan) consisted of a Shimadzu 30 AD liquid chromatography system (LC-30A binary pump, an SIL-30AC autosampler, an SPD-M30A PDA detector, and a CTO-20AC column oven) and an 8050 triple quadrupole mass spectrometer equipped with a heated electrospray ionization source. Data acquisition was performed using the LabSolutions LCMS Ver. 5.6 software (Shimadzu Corp., Kyoto, Japan). Liquid chromatography separations were carried out on a Kinetex 2.6u C18 100 Å column (100 mm × 2.1 mm; i.d. 2.6 μm; Phenomenex, Inc., Torrance, CA, USA) with a flow rate of 0.5 mL/min. The mobile phase consisted of ACN (A) and H_2_O (B), using a gradient elution of 30%–40% A at 0–2 min, 40%–85% A at 2–2.01 min, 85%–90% A at 2.01–6 min, 90% A at 6–10 min and 90%–95% A at 10–12 min. An aliquot (1 μL) of the sample was injected into the LC-MS/MS system and the analysis was carried out at 30 °C.

For mass detection, the acquisition parameters were as follows: drying gas (N_2_) flow rate, 10.0 L/min; nebulizing gas flow rate, 3.0 L/min; heating gas flow rate, 10.0 L/min; interface voltage, 3 kV; detector voltage, 1.8 kV; interface temperature, 300 °C; desolvation temperature, 250 °C; and heat block temperature, 400 °C. The optimized multiple reaction monitor (MRM) parameters including collision energy, Q1 Pre Bias, Q3 Pre Bias, Dwell time, and the MRM transition of the nine standards are listed in Table 7.

### 3.6. Assay Validation

#### 3.6.1. Injection Precision, Linearity of Calibration, Lower Limit of Detection, and Lower Limit of Quantification

The same standard solution was analyzed six times successively to obtain the injection precision and the RSDs of retention times and peak areas were used to evaluate the system suitability. At least nine concentration levels were used to assess the linearity of the nine components. The series concentrations were 0.04, 0.08, 0.16, 0.24, 0.4, 0.8, 1.2, 2.0, 4.0, and 6.0 µg/mL for component **1**; 0.02, 0.04, 0.08, 0.12, 0.2, 0.4, 0.6, 1.0, 2.0, and 3.0 µg/mL for component **2**; 0.02, 0.04, 0.08, 0.12, 0.2, 0.4, 0.6, 1.0, 2.0, and 3.0 µg/mL for component **3**; 0.05, 0.1, 0.2, 0.3, 0.5, 1.0, 1.5, 2.5, 5.0, and 7.5 µg/mL for component **4**; 0.02, 0.04, 0.08, 0.12, 0.2, 0.4, 0.6, 1.0, 2.0, and 3.0 µg/mL for component **5**; 0.02, 0.04, 0.08, 0.12, 0.2, 0.4, 0.6, 1.0, 2.0, and 3.0 µg/mL for component **6**; 0.06, 0.12, 0.24, 0.36, 0.6, 1.2, 1.8, 3.0, 6.0, 9.0, and 24.0 µg/mL for component **7**; 0.08, 0.16, 0.24, 0.4, 0.8, 1.2, 2.0, 4.0, and 6.0 µg/mL for component **8**; and 0.1, 0.2, 0.4, 0.6, 1.0, 2.0, 3.0, 5.0, 10.0, and 15.0 µg/mL for component **9**. The correlation coefficient (*r*) of 0.995 or higher was the acceptance criterion for a calibration curve. The LLOD and LLOQ were determined based on a signal-to-noise of at least 3:1 and 10:1, respectively.

#### 3.6.2. Precision and Stability

Intra-day precision was determined by analyzing six replicates on the same day. Inter-day precision was obtained by duplicating the experiments over three consecutive days.

The normal temperature (25 °C) stability of the sample and reference solutions were tested at 0, 2, 4, 6, 8, 12, 16, and 24 h, respectively. The samples were considered stable if the assay values were within an acceptable deviation from the actual value.

#### 3.6.3. Extraction Recovery

The extraction recoveries of the nine components were determined using the method described in the Pharmacopoeia of the People’s Republic of China [24]. Briefly, 0.25 g of the powder was accurately weighed and added into a conical flask. A certain amount of reference substances was added to make the final contents 80%, 100%, and 120% compared to the actual contents in the ADR samples (0.5 g). The spiked samples were then prepared and measured in accordance with the method described above. For each concentration level, the whole process was repeated three times and the extraction recoveries were calculated using Equation (1).

Recovery (%) = (C − A)/B × 100%(1)

where A is the content of the added reference substance, B is the content of the compound in the ADR sample, and C is the content determined by LC-MS/MS.

## 4. Conclusions

For the first time, a rapid and sensitive UFLC-MS/MS method was established to simultaneously analyze and compare nine new furanocoumarins, including three pairs of isomers (components **1** and **2**, **4** and **5**, and **7** and **8**), in different cultivars of ADR. The developed method was sensitive, accurate, and reproducible. The results from this work can provide essential information for a better understanding of the contents and distribution of the nine coumarins in ADR. In addition, as components **1** and **2** have anti-inflammatory activities, ADR can also be helpful for the usage of ADR in clinical settings.

## Figures and Tables

**Table 1:** Regression equations, correlation coefficients, lower limit of detection (LLOD) and lower limit of quantification (LLOQ) for the nine analytes.

Analyte	Correlation Coefficient (*r*)	Regression Equation ^a^	Linear Range (µg/mL)	LLOD (ng/mL)	LLOQ (ng/mL)
**1**	0.9965	*y* = 4,885,175*x* + 787,567	0.04–6	0.37	1.11
**2**	0.9980	*y* = 10,273,680*x* − 169,101	0.02–3	0.19	0.56
**3**	0.9997	*y* = 1,607,104*x* + 2012	0.02–3	0.56	1.67
**4**	0.9983	*y* = 5,978,103*x* + 845,390	0.05–7.5	0.46	1.39
**5**	0.9994	*y* = 6,547,018*x* + 339,120	0.02–3	0.56	1.67
**6**	0.9998	*y* = 726,813*x* − 19,820	0.02–3	2.78	8.33
**7**	0.9999	*y* = 2,943,619*x* − 82,610	0.06–24	1.67	5.00
**8**	0.9994	*y* = 2,190,557*x* − 3386	0.08–6	1.11	3.33
**9**	0.9996	*y* = 416,419*x* + 29,294	0.1–15	2.78	8.33

^a^ *y* is the peak area, *x* is the concentration of coumarins (µg/mL).

**Table 2:** Precision of the method for the determination of the nine analytes.

Precision	Content ^a^ (µg/g)	RSD (%)	Content ^a^ (µg/g)	RSD (%)	Content ^a^ (µg/g)	RSD (%)
Analyte	**1**		**2**		**3**	
Intra-day ^b^	93.42 ± 1.28	1.37	29.85 ± 0.86	2.87	43.74 ± 1.32	3.02
Inter-day ^c^	91.74 ± 3.04	3.31	28.38 ± 1.39	4.91	42.22 ± 1.97	4.66
Analyte	**4**		**5**		**6**	
Intra-day ^b^	76.73 ± 1.38	1.79	26.20 ± 0.64	2.45	42.61 ± 0.97	2.28
Inter-day ^c^	73.34 ± 3.84	5.23	24.54 ± 1.43	5.82	41.28 ± 1.56	3.78
Analyte	**7**		**8**		**9**	
Intra-day ^b^	116.03 ± 2.91	2.51	59.81 ± 1.19	1.98	223.74 ± 4.64	2.07
Inter-day ^c^	114.37 ± 3.59	3.14	56.45 ± 2.80	4.96	220.01 ± 7.59	3.45

^a^ Mean ± SD (Standard Deviation); ^b^ Six replicates in a single day, *n* = 6; ^c^ Sample analyzed each day on three consecutive days, *n* = 3.

**Table 3:** Stability of the method for the determination of the nine analytes.

Group	Value	1	2	3	4	5	6	7	8	9
**Standard solution**	**Mean**	14,116,389	5,857,693	1,958,105	20,460,626	4,811,869	818,124	10,568,888	3,003,283	2,709,082
	**RSD (%)**	4.16	3.05	4.40	3.15	3.64	5.38	4.30	4.59	5.43
**Sample solution**	**Mean**	15,681,047	5,095,101	2,220,960	16,217,195	3,680,829	945,093	10,855,837	2,447,999	3,064,152
	**RSD (%)**	3.19	5.34	4.32	4.23	5.81	3.83	4.21	4.90	3.76

RSD: Relative Standard Deviation.

**Table 4:** Recoveries of the nine analytes by use of the high-performance liquid chromatography coupled with tandem mass spectrometry (LC-MS/MS) method (*n* = 3).

Analyte	Concentration	Recovery (%)	RSD (%)
**1**	Low	107.45 ± 5.40	5.03
	Medium	101.95 ± 4.48	4.40
	High	95.42 ± 4.52	4.73
**2**	Low	105.11 ± 3.65	3.47
	Medium	88.49 ± 0.59	0.67
	High	100.60 ± 5.48	5.45
**3**	Low	101.85 ± 5.30	5.20
	Medium	103.02 ± 5.58	5.42
	High	100.41 ± 6.58	6.56
**4**	Low	100.85 ± 1.65	1.64
	Medium	96.21 ± 1.54	1.60
	High	96.16 ± 6.09	6.34
**5**	Low	100.67 ± 2.88	2.86
	Medium	86.12 ± 3.69	4.29
	High	90.47 ± 5.19	5.74
**6**	Low	100.97 ± 3.70	3.66
	Medium	107.45 ± 0.37	0.35
	High	104.26 ± 3.07	2.95
**7**	Low	94.44 ± 3.05	3.22
	Medium	99.13 ± 1.71	1.73
	High	102.41 ± 1.87	1.83
**8**	Low	100.14 ± 2.35	2.34
	Medium	92.75 ± 4.34	4.67
	High	102.01 ± 4.46	4.38
**9**	Low	96.06 ± 6.22	6.47
	Medium	95.96 ± 3.62	3.77
	High	92.88 ± 2.99	3.22

**Table 5:** Determination of the nine analytes in different batches of Angelicae Dahuricae Radix.

No.	Amount (μg/g Crude Drug)									
	1	2	3	4	5	6	7	8	9	Total
HBZ201208	95.42	23.34	41.49	78.35	22.51	44.22	122.18	52.30	228.74	708.56
HBZ201508XY	101.89	23.11	22.87	56.10	17.28	11.95	34.04	12.40	169.35	449.00
HBZ201508Y	20.44	4.12	7.25	16.75	2.21	37.02	94.86	25.56	36.79	244.99
HBZ2015S12	333.33	4.11	6.03	30.47	5.88	71.82	411.03	116.24	23.87	1002.79
HBZ2015S14	78.07	2.76	1.77	15.01	5.00	10.47	109.09	51.71	7.36	281.24
CBZ201107	245.82	77.48	41.74	146.84	44.51	21.92	98.50	42.19	303.58	1022.57
CBZ201207	78.57	27.11	11.55	46.38	25.63	8.74	41.45	21.41	79.45	340.27
CBZ201307	87.32	36.41	9.59	49.80	28.92	3.68	21.83	18.32	65.47	321.33
CBZ201407	25.39	8.82	5.02	19.27	8.04	15.65	76.64	57.04	25.04	240.91
CBZ201507	28.65	8.44	3.75	27.98	9.04	11.92	98.44	59.20	14.11	261.53
YBZ201107	26.47	13.64	7.33	29.03	24.44	43.64	249.30	192.26	13.49	599.60
YBZ201207	26.20	6.73	7.40	29.89	9.71	40.32	195.09	112.66	27.52	455.52
YBZ201307	25.62	5.78	7.96	26.54	7.44	37.82	177.86	102.71	33.24	424.97
YBZ201407	25.48	6.60	6.85	28.17	8.62	35.48	179.40	106.75	24.85	422.20
YBZ201507	5.43	2.93	3.22	19.76	7.11	32.72	229.64	218.73	4.11	523.65
QBZ200807	23.33	9.00	4.24	17.74	9.61	7.05	39.54	31.17	17.38	159.07
QBZ201207	3.60	0.86	1.20	3.35	1.38	17.61	46.69	9.28	5.44	89.41
QBZ201307	16.79	3.18	8.44	9.38	1.69	21.11	53.64	20.97	63.59	198.78
QBZ201410	14.28	4.69	7.06	14.81	4.40	22.63	66.24	21.52	27.83	183.46
QBZ201507	86.23	27.21	4.84	44.84	22.38	7.30	56.83	31.29	31.29	312.20

**Table 6:** Samples of 20 batches of *Angelicae dahuricae* Radix analyzed in the present study.

Code No.	Location	Time
HBZ201208	Pan’an City, Zhejiang Province, China	August 2012
HBZ201508XY	Pan’an City, Zhejiang Province, China	August 2015
HBZ201508Y	Yuyao City, Zhejiang Province, China	August 2015
HBZ2015S12	Pan’an City, Zhejiang Province, China	August 2015
HBZ2015S14	Pan’an City, Zhejiang Province, China	August 2015
CBZ201107	Suining City, Sichuan Province, China	July 2011
CBZ201207	Suining City, Sichuan Province, China	July 2012
CBZ201307	Suining City, Sichuan Province, China	July 2013
CBZ201407	Suining City, Sichuan Province, China	July 2014
CBZ201507	Suining City, Sichuan Province, China	July 2015
YBZ201107	Yuzhou City, Henan Province, China	July 2011
YBZ201207	Yuzhou City, Henan Province, China	July 2012
YBZ201307	Yuzhou City, Henan Province, China	July 2013
YBZ201407	Yuzhou City, Henan Province, China	July 2014
YBZ201507	Yuzhou City, Henan Province, China	July 2015
QBZ200807	An’guo City, Hebei Province, China	July 2008
QBZ201207	An’guo City, Hebei Province, China	July 2012
QBZ201307	An’guo City, Hebei Province, China	July 2013
QBZ201410	An’guo City, Hebei Province, China	October 2014
QBZ201507	An’guo City, Hebei Province, China	July 2015

**Table 7:** Optimized MRM parameters of the nine standards in Angelicae Dahuricae Radix.

Analyte	Retention Time (min)	MRM Transition (*m*/*z*)	Dwell Time (ms)	Q1 Pre Bias (V)	Collision Energy (V)	Q3 Pre Bias (V)
		Precursor Ion→Product Ion				
1	5.29	525.2→254.0	10	−40	−40	−30
2	5.52	473.2→203.05	24	−30	−30	−30
3	6.39	523.2→309.05	24	−40	−30	−21
4	6.70	553.2→254.0	10	−40	−40	−30
5	7.03	501.2→203.0	24	−40	−25	−30
6	8.53	577.3→308.95	24	−40	−32	−22
7	9.06	607.3→254.0	10	−40	−43	−30
8	9.60	555.3→203.5	24	−40	−34	−21
9	5.07	495.2→309.05	24	−30	−29	−21

Dwell time: residence time during an acquisition point; Q1 Pre Bias: voltage promotes the ionization of the precursor ion; Q3 Pre Bias: voltage promotes the ionization of the product ion.
